# Distribution of Parasitic Helminths in the Small Intestine of the Red Fox (*Vulpes vulpes*)

**DOI:** 10.3390/pathogens9060477

**Published:** 2020-06-16

**Authors:** Jacek Karamon, Jacek Sroka, Joanna Dąbrowska, Ewa Bilska-Zając, Katarzyna Skrzypek, Mirosław Różycki, Jolanta Zdybel, Tomasz Cencek

**Affiliations:** Department of Parasitology and Invasive Diseases, National Veterinary Research Institute, Partyzantów Avenue 57, 24-100 Puławy, Poland; jacek.sroka@piwet.pulawy.pl (J.S.); Joanna.dabrowska@piwet.pulawy.pl (J.D.); ewa.bilska@piwet.pulawy.pl (E.B.-Z.); katarzyna.skrzypek@piwet.pulawy.pl (K.S.); mrozycki@piwet.pulawy.pl (M.R.); j.zdybel@piwet.pulawy.pl (J.Z.); tcencek@piwet.pulawy.pl (T.C.)

**Keywords:** red fox, helminths, *Echinococcus*, *Toxocara*, *Alaria*, hookworms, *Taenia*, *Toxascaris*, *Mesocestoides*, distribution, intestine, Poland

## Abstract

The aim of the study was to analyze the distribution of the main groups of parasitic helminths within the small intestine of the red fox on the example of animals coming from eastern Poland. Two hundred and sixteen red foxes shot in eastern Poland were used in the investigation. Before examination, each small intestine was divided into three equal parts: anterior (A), middle (M), and posterior (P). Each part was examined separately with the sedimentation and counting technique. Six different types of intestinal parasites were detected: *Alaria alata* (78.7%), *Mesocestoides* spp. (78.2%), hookworms (72.7%), *Taenia* spp. (53.2%), *Toxocara/Toxascaris* (43.1%), and *Echinococcus multilocularis* (18.5%). *Alaria alata* was most often found in A and in the only-A variant. *Taenia* spp. and *Toxocara/Toxascaris* occurred often in A and were the second (after *A. alata*) parasites in terms of frequency occurring in the only-A variant. *Mesocestoides* spp. was most commonly located in M. Parasites with predilection sites located mainly in M and P were *E. multilocularis* and hookworms. In all parasite species, the variant covering the entire intestine (A + M + P) was found in samples with a higher intensity compared to variants limited to one or two fragments. Our investigation, as one of the few of its type, conducted a comprehensive analysis of the distribution of intestinal helminths in the small intestine of the red fox. It showed significant differences in the distribution of parasitic helminths in the small intestine of the red fox. Determining typical predilection sites for parasites in the intestine can be helpful in creating effective diagnostic methods.

## 1. Introduction

Red foxes (*Vulpes vulpes*) are the final hosts of many species of helminths (nematodes, tapeworms, and flukes) parasitizing the small intestine. Among them, there are also zoonotic species of which the developmental stages are dangerous to humans [1,2,3]. In recent years, most attention has been paid to the infection of *E. multilocularis*, for which prevalence in red foxes has increased significantly in Europe in previous decades [4,5,6]. It is a small tapeworm, and in its life cycle, the role of a nonspecific intermediate host can be played by humans, where the larval forms can cause life-threatening alveolar echinococcosis [7]. However, a wide range of other tapeworms have been found in red foxes. Among them, many *Taenia* species have been described, e.g., *Taenia hydatigena, T. crassiceps, T. pisiformis*, *T. polyacantha*, *T. serialis*, and *T. (Hydatigera) teanieaformis* [1,3,8,9,10]. Very common and distributed worldwide are the *Mesocestoisdes* tapeworms that have been found in high percentages in many populations of red foxes [2,3,11,12,13,14]. Moreover, *Dipylidium caninum* was also noted but rather sporadically [8,10]. Among nematodes, roundworms from the family Toxocaridae are common, especially *Toxocara canins* and less frequently *Toxocara cati* and *Toxascaris leonina* [10,15,16,17]. Other popular intestinal nematodes of red foxes are hookworms, most frequently belonging to the *Uncinaria stenocephala* and *Ancylostoma caninum* species [12,18,19]. There are differences in geographical distributions between these two species: *Uncinaria* infections concern colder parts of continents, while the area of *Ancylostoma* occurrence includes countries with a mild climate and relatively high temperatures [20]. Among trematodes, *Alaria alata* can be regarded as the most commonly detected in red fox small intestines. A very high prevalence of this parasite in red foxes (the main definitive host) is reported in regions rich in lake districts, as the life cycle and presence of intermediate hosts is connected with water environments [14,21,22]. Other species of trematodes have been also found (e.g., Echinostomatidae and Opisthorchiidae families) but much less frequently [9]. There are also a few cases of detection of Acanthocephala parasites (e.g., *Macracanthorhynchus* sp.) [9,10].

It must be stressed that free-living red foxes are generally infected with more than one species of intestinal helminths, most often three or four coinfecting parasites [1,14,23]. Therefore, such a rich helminthofauna can cause a set of mutual interactions, which are conditioned by the presence of different parasites together in specific locations in the host intestine [24,25,26]. Data on locations of parasites can be an important basis for studies on interactions between parasites species [25] and between parasites and other organisms (e.g., bacterial intestinal microbiota) [27]. Moreover, knowledge about the distribution of parasites in the intestine is important for the development of effective parasitological diagnostic methods. This enables the selection of appropriate parts of the intestine to efficiently detect specific parasite species. Studies on this issue often referred to *E. multilocularis*, which is associated with the development of diagnostic methods used in monitoring studies [28,29]. For example, the modification of sedimentation technique was focused mainly on the predilection site of *E. multilocularis* tapeworms [29]. 

However, the distribution of helminths in red fox intestine has not been described in detail. In recent years, interesting investigations have been carried out on the distribution of tapeworms as well as *A. alata* in red fox intestine from northwest Poland [21,30]. However, there is a lack of comprehensive studies on the distribution of all helminths in red foxes’ small intestines.

The occurrence of all intestinal helminths in red foxes in Poland were investigated only in selected areas [14,31]. The significant diversity concerning the prevalence of individual parasite species in different regions of Poland were shown [14]. However, there is a lack of data concerning all parasitic helminths in red foxes from the eastern part of the country (Lubelskie Province).

The aim of the study was to analyze the distribution of the main groups of parasitic helminths within the small intestine of the red fox with a sample of animals coming from eastern Poland (Lubelskie Province). In addition, the prevalence of intestinal helminths in red foxes in this region will be assessed.

## 2. Results

### 2.1. Prevalence of Intestinal Helminths in Lubelskie Province

Intestinal helminths were detected in 215 (99.5%) red foxes. Six different genera/species of intestinal parasites were detected, namely *E. multilocularis*, *A. alata*, *Mesocestoide*s spp., *Taenia* spp., hookworms (*Uncinaria/Ancylostoma*), and *Toxocara/Toxascaris*. The most often found parasites were *A. alata*, *Mesocestoides* spp., and hookworms. The parasite with the lowest prevalence was *E. multilocularis*. The highest mean intensity was observed in the case of *E. multilocularis* (above 500 tapeworms per intestine). However, the mean intensity of other parasites did not exceed 90 worms per intestine. Detailed results are presented in Table 1.

### 2.2. Analysis of Location and Distribution of Individual Parasites in the Small Intestine

In the distribution analysis of parasites within the intestine, only animals in which the parasites were found were considered.

***Echinococcus multilocularis***

*Echinococcus multilocularis* tapeworms occurred most frequently in the posterior part of the intestine (P) (92.5%), and this was significantly higher than in the anterior part (A) (57.5%) (*χ*^2^ = 52.38, *p* = 0.0008). There were no significant differences between M and A and between M and P (Table 2). Intensity of *E. mutlilocularis* was similar in each part, and there were no significant differences (Table 3). 

**Figure 1:**
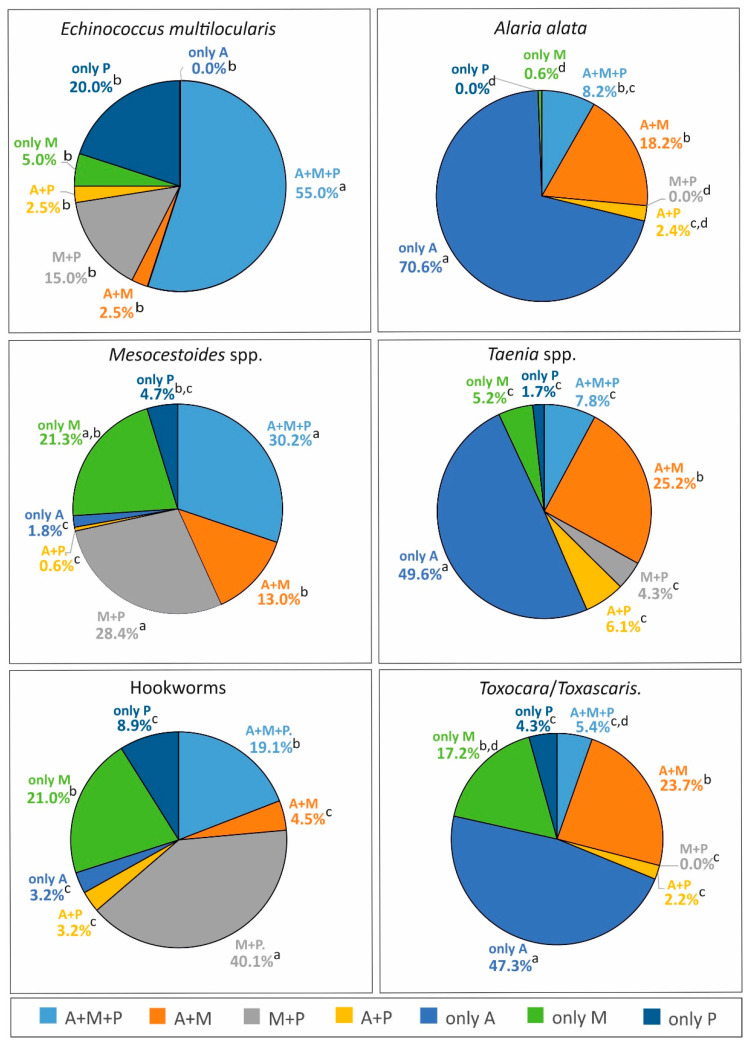
Occurrence of helminth infections in different variants of distribution in the small intestine. Distribution variants: A + M + P (all parts together: anterior, middle, and posterior); A + M (anterior and middle parts together); M + P (middle and posterior together); A + P (anterior and posterior together); only A (only anterior part); only M (only middle part); and only P (only posterior part). ^a,b,c,d^ Different letters in superscript indicate statistically significant differences among variants (*p* < 0.05).

Analysis of *E. multilocularis* variant distributions in intestines showed that the most prevalent was the variant concerning the entire intestine (A + M + P), where 55.0% of foxes infected with *E. multilocularis* indicated this tapeworm in three parts together. Statistically significant differences were demonstrated between A + M + P and all other variants of the distribution: A + M (*χ*^2^ = 24.41, *p* < 0.0001), M + P (*χ*^2^ = 12.36, *p* = 0.0004), A + P (*χ*^2^ = 24.41, *p* < 0.0001), only A (*χ*^2^ = 27.65, *p* < 0.0001), only M (*χ*^2^ = 21.49, *p* < 0.0001), and only P (*χ*^2^ = 9.01, *p* = 0.0027) (Figure 1). A general analysis of the intensity between distribution variants showed statistical differences (*H* = 19.10, *p* = 0.0018). Multiple comparisons indicated significant differences (*p* < 0.0012) only between A + M + P (mean intensity 915.1) and the only-P variant (mean intensity 19.0) (Table 4).

***Alaria alata***

*Alaria alata* were detected in the anterior part of intestine (A) in almost all foxes positive for this infection (99.4%); only one had no *A. alata* flukes in the anterior part. The occurrence in the anterior (A) part was significantly higher than in the other parts: M (*χ*^2^ = 190.77, *p* < 0.0001) and P (*χ*^2^ = 267.38, *p* < 0.0001). Moreover, the number of positive results obtained in the middle part (26.5%) was significantly higher than in the posterior part (10.6%) (*χ*^2^ = 14.20, *p* = 0.0002) (Table 2).

There were significant differences in the intensity of *A. alata* between individual parts of the intestine (*H* = 69.59, *p* < 0.0001). Multiple analyses showed that intensity in the anterior part was significantly higher than in other parts (*p* < 0.0001). There were no differences in intensity between middle and posterior parts (Table 3). 

Analysis of distribution variants indicated that *A. alata* significantly most often occurred only in the anterior part of the intestine (only A) (70.6%) compared to all other variants: A + M + P (*χ*^2^ = 138.39, *p* < 0.0001), A + M (*χ*^2^ = 94.,37, *p* < 0.0001), M + P (*χ*^2^ = 182.38, *p* < 0.0001), A + P (*χ*^2^ = 167.88, *p* < 0.0001), only M (*χ*^2^ = 178.65, *p* < 0.0001), and only P (*χ*^2^ = 182.38, *p* < 0.0001). Next for frequency was the A + M variant, which occurred significantly more often than M + P (*χ*^2^ = 31.94, *p* < 0.0001), A + P (*χ*^2^ = 21.53, *p* < 0.0001), only M (*χ*^2^ = 29.01, *p* < 0.0001), and only P (*χ*^2^ = 31.94, *p* < 0.0001). Moreover, *A. alata* was detected more frequently in the entire intestine (A + M + P) compared to M + P (*χ*^2^ = 14.60, *p* = 0.0001), only M (*χ*^2^ = 10.04, *p* < 0.0015), and only P (*χ*^2^ = 14.60, *p* = 0.0001) (Figure 1).

Statistical comparisons of intensities between distribution variants showed significant differences (*H* = 36.89, *p* < 0.0001) but only between A + M + P (mean intensity 362.5) and the only-A variant (mean intensity 46.0) (*p* < 0.0001) (Table 4).

***Mesocestoides* spp.**

*Mesocestoides* tapeworms were found most frequently in the middle part (92.9%) with a significantly higher incidence than that in the posterior (63.9%) (*χ*^2^ = 41.95, *p* < 0.0001) and anterior parts (45.0%) (*χ*^2^ = 90.64, *p* < 0.0001). The occurrence in the posterior part was also significantly higher than that in the anterior (*χ*^2^ = 12.21, *p* = 0.0005) (Table 2). 

There were significant differences in intensity between parts of the intestine (*H* = 11.08, *p* = 0.0039). Multiple analyses indicated a significantly higher intensity in the middle part compared to the anterior part (*p* = 0.0029) (Table 3). 

There were three dominant distribution variants of *Mesocestoides*, A + M + P (30,2%), M + P (28.4%), and only M (21.3%), without significant differences between them. The first two variants were significantly higher than all four others: A + M + P and A + M (*χ*^2^ = 14.69, *p* = 0.0001), A + M + P and A + P (*χ*^2^ = 54.57, *p* < 0.0001), A + M + P and only A (*χ*^2^ = 48.69, *p* < 0.0001), A + M + P and only P (*χ*^2^ = 36.22, *p* < 0.0001), M + P and A + M (*χ*^2^ = 11.26, *p* = 0.0008), M + P and A + P (*χ*^2^ = 50.51, *p* < 0.0001), M + P and only A (*χ*^2^ = 44.71, *p* < 0.0001), and M + P and only P (*χ*^2^ = 32.55, *p* < 0.0001). However, the only-M variant was significantly higher than only A (*χ*^2^ = 29.68, *p* < 0.0001), only P (*χ*^2^ = 19.05, *p* < 0.0001), and A + P (*χ*^2^ = 35.08, *p* < 0.0001). Moreover, the A + M variant was significantly higher than A + P (*χ*^2^ = 11.26, *p* < 0.0001) and only A (*χ*^2^ = 14.00, *p* = 0.0002) (Figure 1).

Comparisons of intensities between distribution variants of *Mesocestoides* tapeworms showed statistical differences (*H* = 69.11, *p* < 0.0001). Intensity in the A + M + P variant was significantly higher than the only-M and only-P variants (*p* < 0.0001). Additionally, a significant difference in intensity between A + M and only M was observed (*p* = 0.0021) (Table 4).

***Taenia* spp.**

Tapeworms of the *Taenia* genus occurred most often in the anterior part of the intestine (87.0%) and was significantly more frequent than in the middle (43.5%) (*χ*^2^ = 47.92, *p* < 0.0001) and in the posterior parts (20.0%) (*χ*^2^ = 103.61, *p* < 0.0001). Moreover, occurrence in the middle part was significantly higher than that in the posterior part (*χ*^2^ = 14.63, *p* = 0.0001) (Table 2). 

There were no significant differences in intensity of *Taenia* tapeworms between individual parts of the intestine (Table 3).

The most frequent variant of distribution for *Taenia* spp. was only A (49.6%), and it was significantly higher than all other variants: A + M + P (*χ*^2^ = 46.94, *p* < 0.0001), A + M (*χ*^2^ = 13.54, *p* = 0.0002), M + P (*χ*^2^ = 57.43, *p* < 0.0001), A + P (*χ*^2^ = 51.98, *p* < 0.0001), only M (*χ*^2^ = 54.65, *p* < 0.0001), and only P (*χ*^2^ = 66.48 *p* < 0.0001). The presence in the anterior and middle parts together (A + M) was the second most frequent variant; it was significantly higher than A + M + P (*χ*^2^ = 11.38, *p* = 0.0007), M + P (*χ*^2^ = 18.26, *p* < 0.0001), A + P (*χ*^2^ = 14.52, *p* = 0.0001), only M (*χ*^2^ = 16.31, *p* = 0.0001), and only P (*χ*^2^ = 25.20, *p* < 0.0001) (Figure 1).

There were significant differences (*H* = 33.23, *p* < 0.0001) in intensity of *Taenia* between distribution variants. Intensity in the A + M + P variant was significantly higher than the only-A (*p* = 0.0012) and only-M (*p* = 0.0016) variants (Table 4).

**Hookworms (*Uncinaria/Ancylostoma*)**

Hookworms were detected most frequently in the middle (82.8%) and posterior parts (73.2%). Incidences in both parts were significantly higher than in the anterior part (29.9%): *χ*^2^ = 89.21, *p* < 0.0001 and *χ*^2^ = 58.96, *p* < 0.0001, respectively (Table 2).

There were significant differences in intensity of hookworms between parts of the intestine (*H* = 14.92, *p* = 0.0006). Multiple analyses showed that the intensity in the anterior part was significantly lower than in the middle (*p* = 0.0005) and posterior (*p* = 0.0149) parts (Table 3).

Analysis of distribution variants showed that hookworms significantly most often occurred in the M + P variant of distribution (40.1%) than in all other variants: A + M + P (*χ*^2^ = 15.64, *p* = 0.0001), A + M (*χ*^2^ = 55.61, *p* < 0.0001), A + P (*χ*^2^ = 60.99, *p* < 0.0001), only A (*χ*^2^ = 60.99, *p* < 0.0001), only M (*χ*^2^ = 13.50, *p* = 0.0002), and only P (*χ*^2^ = 41.31, *p* < 0.0001). Next for frequency was the only-M (21.0%) and A + M + P (19.1%) variants. The only-M variant occurred significantly more often than A + M (*χ*^2^ = 17.91, *p* < 0.0001), A + P (*χ*^2^ = 21.83, *p* < 0.0001), only A (*χ*^2^ = 21.83, *p* < 0.0001), and only P (*χ*^2^ = 9.03, *p* = 0.0027). Distribution in the entire intestine (A + M + P) was observed more frequently than A + M (*χ*^2^ = 14.83, *p* = 0.0001), A + P (*χ*^2^ = 18.52, *p* < 0.0001), and only A (*χ*^2^ = 18.52, *p* < 0.0001) (Figure 1). 

Statistical comparisons of intensity between distribution variants showed significant differences (*H* = 65.07, *p* < 0.0001). Intensity in the A + M + P variant was significantly higher than the only-A (*p* = 0.0003), only-M (*p* < 0.0001), and only-P (*p* = 0.0009) variants. Moreover, intensity in the M + P distribution was significantly higher than in the only-M variant (*p* < 0.0001) (Table 4).

***Toxocara/Toxascaris***

*Toxocara/Toxascaris* nematodes occurred most often in the anterior part of the intestine (78.5%) significantly more frequent than in the middle (46.2%) (*χ*^2^ = 20.62, *p* < 0.0001) and in the posterior parts (11.8%) (*χ*^2^ = 83.45, *p* < 0.0001). In turn, occurrence in the middle part was significantly higher than in the posterior part (*χ*^2^ = 26.58, *p* = 0.0001) (Table 2). 

There were significant differences in intensity between parts of the intestine (*H* = 12.66, *p* = 0.0018). Multiple analyses showed a significantly higher intensity in the anterior than in the posterior part (*p* = 0.0008) (Table 3).

The most frequent variant of distribution for *Toxocara/Toxascaris* was only A (47.3%), and it was significantly more frequent than all other variants: : A + M + P (*χ*^2^ = 40.01, *p* < 0.0001), A + M (*χ*^2^ = 11.37, *p* = 0.0007), M + P (*χ*^2^ = 55.04, *p* < 0.0001), A + P (*χ*^2^ = 48.55, *p* < 0.0001), only M (*χ*^2^ = 19.29, *p* < 0.0001), and only P (*χ*^2^ = 42.71, *p* < 0.0001). Next in frequency was the A + M (23.7%) and only M (17.2%) variants without significant differences between them. The A + M variant was noted significantly more often than A + M + P (*χ*^2^ = 11.09, *p* = 0.0009), M + P (*χ*^2^ = 22.73, *p* < 0.0001), A + P (*χ*^2^ = 17.27, *p* = 0.0001), and only P (*χ*^2^ = 12.92, *p* = 0.0003). However, the only-M variant occurred significantly more frequently than M + P (*χ*^2^ = 15.39, *p* = 0.0001) and A + P (*χ*^2^ = 10.39, *p* = 0.0013) (Figure 1). 

Statistical comparisons of intensity between distribution variants showed significant differences (*H* = 43.83, *p* < 0.0001). *Toxocara/Toxascaris* intensity noted in the A + M variants was significantly higher than in the only-A (*p* = 0.0007) and only-M (*p* < 0.0001) variants. Moreover, intensity in the A + M + P variant was significantly higher than only M (*p* = 0.0002) (Table 4).

### 2.3. Comparisons of Distributions between Parasite Species

#### 2.3.1. Occurrence in Individual Parts—Comparison between Parasites (Figure 2)

**Anterior part (A).** Statistical analysis showed that, in the anterior part, *A. alata* occurred significantly more frequently than all other parasites: *E. multilocu*laris (*χ*^2^ = 67.13, *p* < 0.0001), *Mesocestoides* (*χ*^2^ = 122.64, *p* < 0.0001), *Taenia* spp. (*χ*^2^ = 17.80, *p* < 0.0001), hookworms (*χ*^2^ = 172.62, *p* < 0.0001), and *Toxocara/Toxascaris* (*χ*^2^ = 33.01, *p* < 0.0001). Next for frequency was *Taenia* spp. and *Toxocara/Toxascaris* detected in statistically similar percentages of infected animals. *Taenia* spp. was significantly more often found than *E. multilocularis* (*χ*^2^ = 15.72, *p* = 0.0001), *Mesocestoides* spp. (*χ*^2^ = 51.19, *p* < 0.0001), and hookworms (*χ*^2^ = 86.90, *p* < 0.0001). *Toxocara/Toxascaris* was more frequent than *Mesocestoides* spp. (*χ*^2^ = 27.49, *p* < 0.0001) and hookworms (*χ*^2^ = 55.17, *p* < 0.0001).

*Echinococcus, Mesocestoides* spp., and hookworms had the lowest percentages. Moreover, *E. multilocularis* occurred more often than hookworms (*χ*^2^ = 10.57, *p* = 0.0011). 

**Middle part (M).** In the middle part, three parasite species (*E. multilocularis*, *Mesocestoides,* and hookworms without significant differences between them) were most often found; all of them occurred significantly more frequently in this part than other parasites. *E. multilocularis* differed significantly from *A. alata* (*χ*^2^ = 37.68, *p* < 0.0001), *Taenia* spp. (*χ*^2^ = 14.46, *p* = 0.0001), and *Toxocara/Toxascaris* (*χ*^2^ = 11.63, *p* = 0.0007). *Mesocestoides* spp. differed from *A. alata* (*χ*^2^ = 155.30, *p* < 0.0001), *Taenia* spp. (*χ*^2^ = 84.58, *p* < 0.0001), and *Toxocara/Toxascaris* (*χ*^2^ = 72.31, *p* < 0.0001). Hookworms were more frequent than *A. alata* (*χ*^2^ = 104.12, *p* < 0.0001), *Taenia* spp. (*χ*^2^ = 45.86, *p* < 0.0001), and *Toxocara/Toxascaris* (*χ*^2^ = 36.64, *p* < 0.0001). Moreover, *A. alata* was found in the middle part significantly less often than *Toxocara/Toxascaris* (*χ*^2^ = 10.55, *p* = 0.0012) and *Taenia* spp. (*χ*^2^ = 8.93, *p* = 0.0028).

**Posterior part (P).** The proportion (and significant differences) between parasites in the posterior part was similar to that in the middle; the same three parasites species were dominant. *E. multilocularis* differed significantly from *A. alata* (*χ*^2^ = 108.19, *p* < 0.0001), *Taenia* spp. (*χ*^2^ = 62.72, *p* < 0.0001), and *Toxocara/Toxascaris* (*χ*^2^ = 75.46, *p* < 0.0001). *Mesocestoides* spp. differed from *A. alata* (*χ*^2^ = 103.16, *p* < 0.0001), *Taenia* spp. (*χ*^2^ = 53.09, *p* < 0.0001), and *Toxocara/Toxascaris* (*χ*^2^ = 65.63, *p* < 0.0001). Hookworms were more frequent than *A. alata* (*χ*^2^ = 132.81, *p* < 0.0001), *Taenia* spp. (*χ*^2^ = 73.30, *p* < 0.0001), and *Toxocara/Toxascaris* (*χ*^2^ = 88.14, *p* < 0.0001). Additionally, a significant difference between *E. multilocularis* and *Mesocestoides* appeared: the former occurred significantly more frequently (*χ*^2^ = 11.14, *p* = 0.0008). 

**Figure 2:**
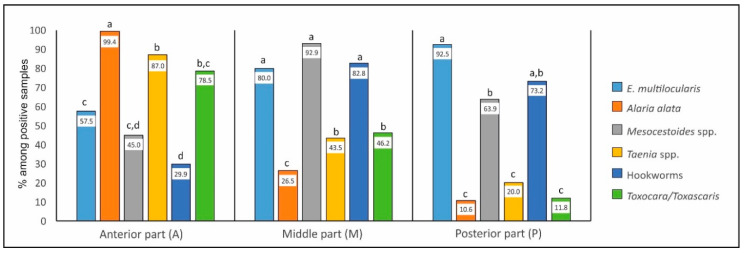
Comparison between occurrence of parasites in individual parts of the small intestine (percentages of samples among positive foxes containing parasites in individual parts of intestine). ^a,b,c,d^ Different letters above the chart bars indicate statistically significant differences among parasites in the same part of the intestine (*p* < 0.05).

Details are showed in Figure 2.

#### 2.3.2. Comparison of Distribution Variants between Individual Parasite Species (Table 5)

**A + M + P variant.** The variant of distribution in the entire intestine was most often observed in the case of *E. multilocularis* infection (55.0%). It was significantly more frequently noted in this tapeworm than in cases of *A. alata* (*χ*^2^ = 49.86, *p* < 0.0001), *Mesocestoides* (*χ*^2^ = 8.77, *p* = 0.0031), *Taenia* spp. (*χ*^2^ = 38.38, *p* < 0.0001), hookworms (*χ*^2^ = 19.33, *p* < 0.0001), and *Toxocara/Toxascaris* (*χ*^2^ = 39.56, *p* < 0.0001). *Mesocestoides* was the next parasite most widely distributed in the intestine (30.2%); the A + M + P variant significantly more often occurred in *Mesocestoides* infection than in infections of *A. alata* (*χ*^2^ = 24.93, *p* < 0.0001), *Taenia* spp. (*χ*^2^ = 19.20, *p* < 0.0001), and *Toxocara/Toxascaris* (*χ*^2^ = 20.51, *p* < 0.0001).

**A + M variant.** This variant was observed at a similar frequency in most parasite species. Only hookworm infections were noted less frequently than *Taenia* spp. (*χ*^2^ = 23.13, *p* < 0.0001) and *Toxocara/Toxascaris* (*χ*^2^ = 19.16, *p* < 0.0001).

**M + P variant.** This range of distribution was noted most frequently in three species: hookworms, *Mesocestoides* spp., and *E. multilocularis*; there were no statistical differences between them. However, all other species were observed significantly less often. The results of statistical comparisons with significant differences were as follows: differences between hookworms and *A. alata* (*χ*^2^ = 81.94, *p* < 0.0001), *Taenia* spp. (*χ*^2^ = 43.43, *p* < 0.0001), and *Toxocara/Toxascaris* (*χ*^2^ = 47.78, *p* < 0.0001); between *Mesocestoides* spp. and *A. alata* (*χ*^2^ = 53.94, *p* < 0.0001), *Taenia* spp. (*χ*^2^ = 24.53, *p* < 0.0001), and *Toxocara/Toxascaris* (*χ*^2^ = 30.47, *p* < 0.0001); and between *E. multilocularis* and *A. alata* (*χ*^2^ = 21.12, *p* < 0.0001) and *Toxocara/Toxascaris* (*χ*^2^ = 11.33, *p* = 0.0008). However, no difference was found between *E. multilocularis* and *Taenia* spp.

**A + P variant.** This combination was observed very rarely in the case of each parasite (from 0.6% to 6.1%), and no statistical differences between species were noted.

**Only-A variant**. This variant was observed most frequently in *A. alata* infection (70.6% of *A. alata* positive intestines had these flukes localized only in the anterior part), significantly more often than in case of all other parasites: *E. multilocu*laris (*χ*^2^ = 63.03, *p* < 0.0001), *Mesocestoides* (*χ*^2^ = 170.62, *p* < 0.0001), *Taenia* spp. (*χ*^2^ = 12.88, *p* = 0.0003), hookworms (*χ*^2^ = 154.19, *p* < 0.0001), and *Toxocara/Toxascaris* (*χ*^2^ = 13.88, *p* = 0.0002). Next, two parasite infections observed very often in this variant of distribution (in similar percentage) were *Taenia* spp. (49.6%) and *Toxocara/Toxascaris* (47.3%). They were noted significantly more often than *E. multilocu*laris (*χ*^2^ = 29.26, *p* < 0.0001; *χ*^2^ = 26.18, *p* < 0.0001, respectively), *Mesocestoides* (*χ*^2^ = 90.95, *p* < 0.0001; *χ*^2^ = 81.44, *p* < 0.0001, respectively), and hookworms (*χ*^2^ = 78.52, *p* < 0.0001; *χ*^2^ = 69.33, *p* < 0.0001, respectively).

**Only-M variant.** Comparison between species showed that, in this variant, *Mesocestoides* spp., hookworms, and *Toxocara/Toxascaris* were observed the most frequently (at similar percentages). These three species were significantly more frequently found in the only-M variant than *A. alata* (*χ*^2^ = 35.30, *p* < 0.0001; *χ*^2^ = 34.41, *p* < 0.0001; *χ*^2^ = 24.77, *p* < 0.0001, respectively). Moreover, *Mesocestoides* spp. and hookworms occurred only in the middle part significantly more often than *Taenia* spp. (*χ*^2^ = 12.80, *p* = 0.0003; *χ*^2^ = 12.24, *p* = 0.0005, respectively).

**Only-P variant.** This variant was observed most frequently in *E. multilocularis* infections. Among samples with *E. multilocularis,* 20% contained these tapeworms only in the posterior part. It was noted more often in *E. multilocularis* than in *A. alata* (*χ*^2^ = 30.10, *p* < 0.0001), *Mesocestoides* (*χ*^2^ = 8.61, *p* = 0.0033), and *Taenia* spp. (*χ*^2^ = 13.51, *p* = 0.0002) infections. Moreover, hookworms were found in the only-P variant significantly more frequently than *A. alata* (*χ*^2^ = 13.74, *p* = 0.0002). 

Details are showed in Table 5.

#### 2.3.3. Relationships between the Intensity of Different Parasites Existing in the Same Intestines

Spearman rank correlation test showed statistically significant (*p* < 0.05), positive correlations between intensities of *Mesocestoides* spp. and *E. multilocularis* (r_s_ = 0.151497), of *Mesocestoides* spp. and *Taenia* spp. (r_s_ = 0.264254), of *Mesocestoides* spp. and hookworms (r_s_ = 0.171528), and of *A. alata* and hookworms (r_s_ = 0.135900). 

## 3. Discussion

### 3.1. Prevalence

In our study, the most prevalent parasites were *A. alata*, *Mesocestoides* spp., and hookworms (with similar high mean prevalence from 73% to 79%) followed by *Taenia* spp., *Toxocara,* and *E. multilocularis*. 

Prevalence of *A. alata* strongly depends on the region and presence of water reservoirs (necessary for the development of intermediate hosts). It is significantly visible in a previous study carried out in Poland [14] where, in northern provinces with a high number of lakes, the prevalence reached more than 90%, while in the southern part of the country (with smaller surface water area), prevalence was only about 20%. The relatively high prevalence (79%) in Lubelskie Province obtained in our study was expected because, in this region, some lake districts are present. Similar correlations connected with regions were observed in different studies conducted in Poland [21,23,33]. In other parts of Europe, the percentage of *A. alata* infected foxes differs depending on the investigation (and region) from very high [3,34], moderate, and low [1,8,12,18,19,35] to areas where these trematodes were not detected [17,36,37].

The second parasite in our study characterized by high prevalence (78.2%) is *Mesocestoides* spp. The percentage of infected foxes is similarly high as in the study conducted recently in different regions of Poland [14] (mean prevalence 84.1%), and it was especially close to the prevalence in the southwest region (77.3%) [14] directly bordering the area investigated in the present study. High and medium prevalence of *Mesocestoides* spp. in red foxes were observed also in other Polish regions (41.3–76%) [23,30,31,38] and in many European countries [2,3,9,11,12,36]. However, there are areas in Europe where these tapeworms in foxes are found very rarely or not at all [1,17,18]. Genetic analysis of *Mesocestoides* isolated in Poland [39,40] allowed us to assume that tapeworms detected in our study mostly belonged to the *Mesocestoides litteratus* species. 

The third most highly prevalent parasites in our study were hookworms (72.2%). Our recent studies showed a similar prevalence in other Polish regions [14,23]. Although, in earlier studies, in Poland [31,41], the percentage of infected animals was significantly lower, which probably was caused by using less sensitive methods for examination of intestines. In our investigation, species of these helminths were not identified, but previous studies carried out in Poland [20,31,41,42] and Europe [1,17,18,19] may suggest that the vast majority of them belonged to *Uncinaria stenocephala*.

More than half of the red foxes in Lubelskie Province (53.2%) were infected with *Taenia* spp. tapeworms. This result is similar to the prevalence observed in southern, northern, northeastern, and southeastern Poland [14]. Lower percentages (28.4% and 29.7%) were observed in northwestern [30] and in central Poland [31], respectively. It may indicate the occurrence of different intermediate hosts in individual regions. It is hard to conclude definitely because we analyzed all tapeworms from *Taenia* genus together without distinguishing species (e.g., *T. crassiceps, T. pisiformis, T. polyacantha, T. hydatigena,* and *T. taeniaeformis*) [1,8,16] which have different lifecycles with various intermediate hosts. In other European countries, *Taenia* spp. tapeworms were reported frequently at different prevalence (from 8% to 62%) [3,8,12,13,16,17].

Because *T. canis* and *T. leonina* are difficult to distinguish in routine microscopic examination, they were analyzed together. However, based on experience form our previous study [14], we can assume that the majority (approximately 80%) of these positive samples belonged to the *Toxocara canis* species. In the present study, the prevalence of these nematodes was similar to results obtained in other Polish regions [14,31,38,41] and Europe [1,2,3,8,10,13,16,19,37].

The parasite with the lowest prevalence was *E. multilocularis* (18.5%). It should be noted that the samples analyzed in this work were part of the material described previously in the context of *E. multilocularis* in the Lubelskie Province [32]. This prevalence is similar to the mean prevalence in red foxes observed in Poland (16.5%) [43]. However, in Poland, there are large variations in the percentage of infected foxes related to geographical location: in the eastern half of the country, it ranges from 18% to 50%, and in the west, it fluctuates around a low percent. However, in recent years, some dynamic changes have been observed related to the gradual increase in prevalence in some western provinces in which this infection was previously at a very low level [44]. The latest data from Zachodniopomorskie Province [30] indicated that infections in the northwestern part of Poland have remained around 5%. In Europe, the situation concerning the prevalence of *E. multilocularis* in red foxes is different and ranges from countries free from this parasite to highly endemic regions [4,6,45,46]. 

### 3.2. Location and Distribution in the Small Intestine

The presented studies showed significant differences in the distribution of various species of parasites in the small intestine of red foxes.

In the anterior part of the intestine (A), *A. alata* flukes were most often found. Practically every *A. alata*-positive sample (99%) had these parasites in the anterior part, whereas in the M and P parts, they were found frequently less often (26.5% and 10.6%). Among other helminths, *A. alata* was significantly most frequently found in A. The special predilection of this fluke to the anterior part of the small intestine is confirmed by the analysis of distribution variants, which showed that, in the vast majority of *A. alata*-positive samples (71%), the parasite was detected only in this part (only-A variant). Similarly, high percentages of *A. alata*-positive samples in the duodenum were found in studies conducted in western Poland [21], where approximately 90% of positive samples (50% of all examined) showed the presence of this trematode in the duodenum. However, in these studies, a similarly high percentage was found in the jejunum. This probably does not indicate a different distribution of the parasites in the intestine, but rather, it is associated with a different division of the intestine in this study. Namely, our anterior part (A) in addition to the duodenum also included the anterior part of the jejunum. The close predilection to the anterior part of the intestine is also evidenced by the ten times higher mean intensity of these parasites in this part than in the middle and posterior ones. This sheds new light on the results published by Tylkowska et al (2018) [21], where the intensity in the jejunum was significantly higher than in the duodenum. However, in analyzing these results together, while taking into account the fact that the anterior one-third (A) in our study also included the beginning of the jejunum, one can be tempted to more accurately determine the predilection site of *A. alata*, which is probably the fragment covering the end of the duodenum and the beginning of the jejunum [21].

In our investigation, similar proportions of locations in three parts of intestines were observed in the cases of *Taenia* spp. and *Toxocara/Toxascaris*. Both parasites occurred more often in the anterior part of intestine than in the middle and posterior parts. Additionally, occurrence in the middle part was significantly higher than in the posterior part. This indicates a predilection for the anterior part of the intestine, but not so clearly visible as in the case of *A. alata*. In addition, both *Taenia* spp. and *Toxocara/Toxascaris* are the second (after *A. alata*) parasites in terms of frequency occurring only in part A (only-A variant). The results obtained in northwestern Poland [30] regarding the distribution of tapeworms roughly coincide with ours. Namely, these parasites were most often detected in the duodenum, slightly less often in the jejunum, and the least often in the ileum (the final part of the small intestine). The difference was only in intensity; our studies did not show any differences between the sections, while in the studies of Tylkowska et al (2019) [30], a significantly higher number of tapeworms was found in the jejunum. Perhaps this difference is also caused by slightly different lines dividing the intestine into three parts [30].

In our investigation, the most common parasites located in the middle part of the intestine (M) are tapeworms of the genus *Mesocestoides*. Almost all *Mesocestoides*-positive (92.9%) samples contained these tapeworms in this part, while in the remaining parts, the percentages were 45.0% (A) and 63.9% (P). 

Moreover, our study showed that the infection intensity in M was significantly higher than in A but did not differ from the intensity in the posterior part (P). A similar distribution was observed by Tylkowska et al. (2019) [30]: in the jejunum, *Mesocestoides* were found twice as frequently and at a much higher intensity compared to the duodenum and ileum. If we consider the differences in the division of the intestine, where in our studies the final fragment of the jejunum was included in the posterior part (P) and its initial segment in anterior part (A), we can conclude that these parasites occupy their typical location, mainly the entire jejunum with a shift towards its posterior part.

In our study, parasites with predilection sites located mainly in the middle and posterior sections of the small intestine (M and P) were *E. multilocularis* and hookworms. In the case of hookworms, this predilection is also emphasized by a significantly higher intensity in M and P compared to A. Moreover, hookworms were most often found in the M + P variant (40%) and only-M variant (21%). Higher preferences of *E. multilocularis* to the posterior part (P) of the small intestine are indicated by the highest percentage of positive samples, in which tapeworms were found only in P (only-P variant). In the study conducted in northwestern Poland [30], *E. multilocularis* was found only in the jejunum and ileum (in a similar percentage). The posterior part of the small intestine is considered a predilection site for *E. multilocularis* [28,29,32]. However, the occurrence of these tapeworms in the anterior parts is associated mainly with the spread of intensive infections throughout the entire intestine. This also confirms the investigations on the modified sedimentation method (segmental sedimentation and counting technique, SSCT) [29], where, only in one intestine among 117 positive intestines (each divided into five parts), *E. multilocularis* was found only in the anterior part. This probably is due to the early infection stage, just after ingestion of larvae, when tapeworms can only be observed in the anterior part of the intestine before they reach their typical location [29].

In our investigation, a positive correlation can be observed between the intensity of infection and the degree of spread of parasites in the intestine. When parasites occur at high numbers, they spread beyond their predilection sites. In all parasite species, the variant covering the entire length of the intestine (A + M + P) was found in samples with a higher intensity compared to variants limited to one or sometimes two fragments of intestine. Variant A + M + P was most frequently found in *E. multilocularis* infection: in more than half of *E. multilocularis*-positive samples, these tapeworms were found in all three parts together.

Comparison of the intensity of different parasitic infections in the same intestines showed no significantly negative correlation between coinfecting species. This indicates that, between the tested intestinal parasite species, there is no significant competition leading to competitor reduction. This may be associated with different intestinal distributions and other predilection sites. However, the reason for this can be a specific adaptation to within-host competition and achieving a balance between individual parasite species, which allows them to coexist in the same host [47]. It may suggest that antagonistic interactions between intestinal helminths of the red foxes play no or minor role in their coexistence. The similar lack of antagonistic interactions between cooccurring nematodes was demonstrated in wild rodents populations [25]. The authors of this paper concluded that this type of interaction is observed relatively often in experimental infections; however, in wild animal populations, it occurs very rarely [24,25].

On the other hand, positive correlations regarding the intensity of *Mesocestoides* and other tapeworms as well as hookworms and *A. alata* may be the result of a higher susceptibility of individual foxes to parasitic infections in general, e.g., caused by general immunosuppression. Another reason for a positive correlation in the intensity of coinfecting parasites, having in their life cycle intermediate hosts (*Mesocestoides*, *Taenia*, and *E. multilocularis*), may be the high hunting activity of individual foxes. However, these are only assumptions because we did not have additional data such as, e.g., age, health condition, or eating behavior [48]. The similar positive correlation was observed in the study conducted on rabbits where intensity of *Graphidium strigosum* was higher in host coinfected with another nematode (*Trichostrongulus retortaeformis*) than single-infected hosts [26].

## 4. Materials and Methods

**Figure 3:**
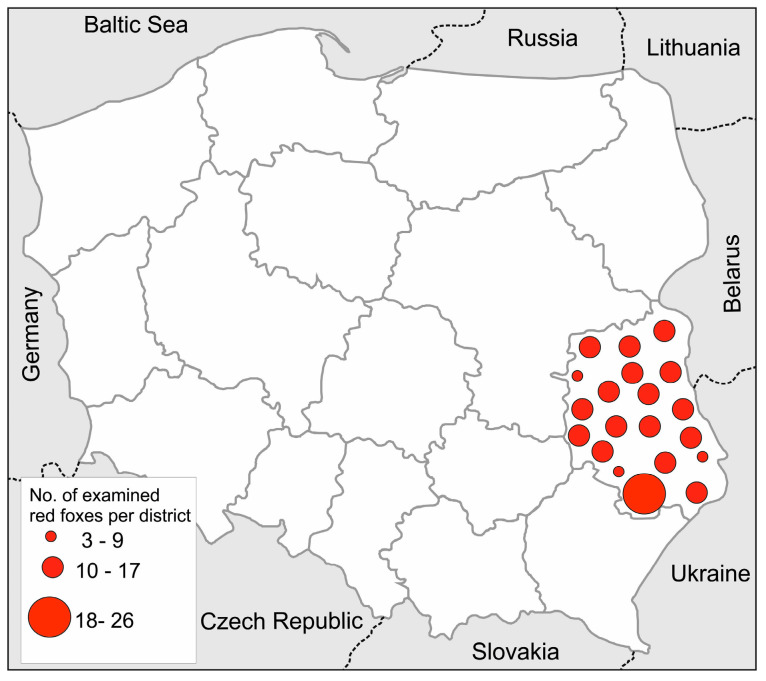
Distribution of the red foxes used in the investigation: The number of foxes obtained in individual districts (powiats) of Lubelskie Province were schematically presented by circles of different sizes.

**Figure 4:**
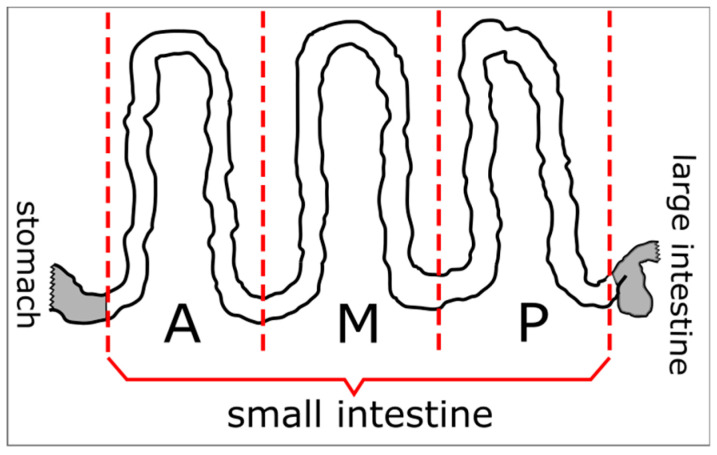
Schematic presentation of the division of the small intestine into three parts: A, anterior; M, middle; and P, posterior.

Two hundred and sixteen red foxes shot in Lubelskie Province (eastern Poland) (NUTS 2 code: PL81) (Figure 3) from 2007 to 2008 were used in the investigation. Foxes were obtained during an official survey concerning the effectiveness of an anti-rabies vaccination. These samples were part of the material obtained and analyzed previously in Lubelskie Province in the frame of surveillance only in the context of *E. multilocularis* [32]. The investigation materials were the small intestines. The samples were stored for at least two weeks at a temperature lower than −70 °C before examination to inactivate dangerous tapeworm (*Echinococcus*) eggs. Before examination, each small intestine was divided into three equal parts (anterior—A, middle—M, and posterior—P) (Figure 4). Each part was separately prepared and examined with the use of the sedimentation and counting technique (SCT) [49,50] to detect parasitic helminths.

**Statistical analysis**. In the distribution analysis of parasites within the intestine, only animals in which the parasites were found were considered. Differences in the percentages of the individual infections among different parts of intestines or different variants of distribution were estimated by a chi-square test (or chi-square with Yates correction) with Bonferroni correction for multiple comparisons. Confidence intervals of the percentages of infected foxes were calculated according to the method described by Newcombe [51]. The distribution of quantitative variables was tested by the Shapiro–Wilk test, and the normality hypothesis of the data was rejected. Differences between multiple groups of the quantitative variables (intensity of infections) were determined by the Kruskal–Wallis test with Bonferroni correction. The relationship between the intensity of individual parasite species was calculated with Spearman’s rank-order correlation. The differences in all analyses were considered statistically significant when *p* < 0.05. Statistical analyses were performed using Statistica 9.1 Stat Soft.

## 5. Conclusions

Our investigation, as one of the few of its type, conducted a comprehensive analysis of the distribution of parasitic intestinal helminths in the small intestine of red foxes. Significant differences in distributions between particular species were found. Determining typical predilection sites for parasites in the intestine not only is of cognitive value but also, in practice, can be helpful in creating effective diagnostic methods. Moreover, our investigation presented for the first time the prevalence and intensity of intestinal helminths in red foxes in this region of Poland.

## Figures and Tables

**Table 1:** Prevalence and intensity of intestinal helminths in red foxes in Lubelskie Province (Poland).

	% of Positive Foxes (95%, CI)	Mean Intensity (CV%)
*Echinococcus multilocularis* ^a^	18.5 (13.9–24.2)	507.3 (92.6)
*Alaria alata*	78.7 (72.8–83.6)	82.2 (145.6)
*Mesocestoides* spp.	78.2 (72.3–83.2)	85.0 (143.3)
*Taenia* spp.	53.2 (46.6–59.8)	8.9 (78.8)
Hookworms	72.7 (66.4–78.2)	9.7 (70.9)
*Toxocara/Toxascaris*	43.1 (36.6–49.7)	4.8 (110.5)

^a^ Data concerning *E. multilocularis* were a part of a study published earlier [32]. Abbreviations: CV, coefficient of variation; CI, confidence interval.

**Table 2:** Comparison of the occurrence of parasites in different parts of the intestine (A—anterior, M—middle, and P—posterior).

	Number of Positive Foxes	Percentage of Samples (Among Positive Foxes) Containing Parasites in Individual Parts of Intestines		
		A	M	P
***Echinococcus*** *multilocularis*	40	57.5 ^a^	80.0 ^a,b^	92.5 ^b^
*Alaria alata*	170	99.4 ^a^	26.5 ^b^	10.6 ^c^
*Mesocestoides* spp.	169	45.0 ^a^	92.9 ^b^	63.9 ^c^
*Taenia* spp.	115	87.0 ^a^	43.5 ^b^	20.0 ^c^
Hookworms	157	29.9 ^a^	82.8 ^b^	73.2 ^b^
*Toxocara/Toxascaris*	93	78.5 ^a^	46.2 ^b^	11.8 ^c^

^a,b,c^ Different letters in superscript indicate statistically significant differences among parts of intestines (*p* < 0.05).

**Table 3:** Comparison of intensity of parasite infections between parts of the small intestine.

	Mean Intensity in Different Parts of Intestines (CV%)		
	A	M	P
***Echinococcus*** *multilocularis*	121.9 ^a^ (311.5)	199.7 ^a^ (126.2)	300.0 ^a^ (211.3)
*Alaria alata*	80.0 ^a^ (168.4)	8.4 ^b^ (190.7)	3.8 ^b^ (106.0)
*Mesocestoides* spp.	29.6 ^a,c^ (317.0)	54.1 ^b^ (249.9)	33.6 ^b,c^ (351.4)
*Taenia* spp.	7.0 ^a^ (112.5)	5.3 ^a^ (117.0)	2.9 ^a^ (108.3)
Hookworms	2.5 ^a^ (105.5)	5.8 ^b^ (119.1)	5.6 ^b^ (137.0)
*Toxocara/Toxascaris*	4.4 ^a^ (148.2)	2.6 ^b^ (108.7)	1.4 ^b^ (49.4)

^a,b,c^ Different letters in superscript indicate statistically significant differences in parasite intensity among parts of intestines (*p* < 0.05). Abbreviations: CV, coefficient of variation; A, anterior; M, middle; and P, posterior.

**Table 4:** Comparison of intensity of helminth infections between different variants of distribution in the small intestine.

	Mean Intensity in Different Distribution Variants (95%, CI)						
	A + M + P	A + M	M + P	A + P	Only A	Only M	Only P
***Echinococcus*** *multilocularis*	915.1 ^a^ (124.5)	24.0 ^a,b^	72.8 ^a,b^ (151.9)	9.0 ^a,b^	na	15.5 ^a,b^ (123.2)	19.0 ^b^ (195.6)
*Alaria alata*	362.5 ^a^ (76.3)	99.2 ^a,b^ (119.8)	na	70.8 ^a,b^ (161.5)	46.0 ^b^ (161.0)	3.0 ^a,b^	na
*Mesocestoides* spp.	178.6 ^a^ (201.1)	116.8 ^a,b^ (256.9)	45.5 ^a,b^ (146.2)	2.0 ^a,b^	2.0 ^a,b^ (86.6)	13.6 ^b^ (196.5)	3.4 ^b^ (119.5)
*Taenia* spp.	19.6 ^a^ (53.9)	12.4 ^a,b^ (85.4)	14.6 ^a,b^ (74.7)	9.4 ^a,b^ (86.8)	6.0 ^b^ (121.4)	2.2 ^b^ (73.9)	3.5 ^a,b^ (101.0)
Hookworms	18.9 ^a^ (88.3)	6.3 ^a,b^ (49.2)	10.9 ^a,b^ (92.1)	3.2 ^a,b^ (40.7)	1.6 ^b^ (83.9)	2.6 ^b^ (70.8)	7.3 ^b^ (193.8)
*Toxocara/Toxascaris*	14.4 ^a^ (61.4)	7.5 ^a^ (79.7)	na	25.5 ^a,b^ (130.3)	3.0 ^a,b^ (111.6)	1.4 ^b^ (64.4)	1.8 ^a,b^ (54.7)

^a,b^ Different letters in superscript indicate statistically significant differences in parasite intensity among parts of intestines (*p* < 0.05).

**Table 5:** Comparison of distribution variants between individual parasite species.

	Percentage of Samples (among Positive Foxes) Containing Parasites in Individual Variants of Distribution					
Variants of Distribution	*Echinococcus multilocularis*	*Alaria alata*	*Mesocestoides* spp.	*Taenia* spp.	Hookworms	*Toxocara/ Toxascaris*
A + M + P	55.0 ^a^	8.2 ^b^	30.2 ^c^	7.8 ^b^	19.1 ^b,c^	5.4 ^b^
A + M	2.5 ^a^	18.2 ^a^	13.0 ^a^	25.2 ^a^	4.5 ^b^	23.7 ^a^
M + P	15.0 ^a,c^	0.0 ^b^	28.4 ^a^	4.3 ^b,c^	40.1 ^a^	0.0 ^b^
A + P	2.5 ^a^	2.4 ^a^	0.6 ^a^	6.1 ^a^	3.2 ^a^	2.2 ^a^
only A	0.0 ^c^	70.6 ^a^	1.8 ^c^	49.6 ^b^	3.2 ^c^	47.3 ^b^
only M	5.0 ^a^	0.6 ^b^	21.3 ^a^	5.2 ^b^	21.0 ^a^	17.2 ^a^
only P	20.0 ^a^	0.0 ^c^	4.7 ^b,c^	1.7 ^b,c^	8.9 ^a,b^	4.3 ^a,b,c^

^a,b,c^ Different letters in superscript in the same row indicate statistically significant differences among variants (*p* < 0.05). Abbreviations: A + M + P, all parts together (anterior, middle, and posterior); A + M, anterior and middle parts together; M + P, middle and posterior together; A + P, anterior and posterior together; only A, only anterior part; only M, only middle part; and only P, only posterior part.
